# Cytomegalovirus Infection of the Anterior Segment: Corneal Endotheliitis and Secondary Glaucoma

**DOI:** 10.3390/pathogens15040371

**Published:** 2026-03-31

**Authors:** Fan Liu, Yaru Zou, Mingming Yang, Jing Zhang, Kyoko Ohno-Matsui, Koju Kamoi

**Affiliations:** Department of Ophthalmology & Visual Science, Graduate School of Medical and Dental Sciences, Institute of Science Tokyo, Tokyo 113-8510, Japan; liufan0417@outlook.com (F.L.); alicezouyaru519@gmail.com (Y.Z.); yangmm-12@outlook.com (M.Y.); zhangj.c@foxmail.com (J.Z.); k.ohno.oph@tmd.ac.jp (K.O.-M.)

**Keywords:** cytomegalovirus, anterior segment infection, corneal endotheliitis, intraocular pressure, secondary glaucoma

## Abstract

Cytomegalovirus (CMV) infection of the anterior segment is increasingly recognized as an important cause of corneal endotheliitis and secondary glaucoma, even in immunocompetent individuals. CMV corneal endotheliitis typically presents with coin-shaped or linear keratic precipitates (KPs), corneal edema, mild anterior chamber inflammation, and recurrent intraocular pressure (IOP) elevation; persistent or episodic ocular hypertension may progress to glaucomatous optic neuropathy if inadequately treated. Definitive diagnosis relies on aqueous humor polymerase chain reaction (PCR) testing for CMV DNA, supported by adjunctive imaging including specular microscopy, anterior segment optical coherence tomography (AS-OCT), and in vivo confocal microscopy (IVCM). Management requires a comprehensive strategy integrating antiviral therapy, anti-inflammatory treatment, and appropriate IOP control. Topical or systemic ganciclovir remains the cornerstone, while refractory disease may necessitate surgical intervention. Older age and male sex, host immune status, prolonged or recurrent CMV infection, and pre-existing ocular conditions are major risk factors for progression and poor outcomes. The pathogenesis of secondary glaucoma is thought to involve both direct viral cytopathic effects and inflammation-mediated damage to the trabecular meshwork (TM), resulting in impaired aqueous outflow. Therefore, early recognition, accurate diagnosis, and effective treatment are essential to prevent corneal decompensation and permanent vision loss.

## 1. Introduction

Cytomegalovirus (CMV) is a ubiquitous β-herpesvirus that establishes lifelong latency in the host after primary infection. Although CMV primarily causes severe symptoms in immunocompromised individuals, growing evidence suggests that it can also lead to clinically significant anterior segment disease in immunocompetent patients [1,2,3,4]. Multiple anterior segment structures can be infected, with evidence of viral presence in the corneal endothelium, iris, and trabecular meshwork (TM) [5]. 

Among the diverse ocular manifestations of CMV infection, corneal endotheliitis represents a distinct clinical entity characterized by inflammation and dysfunction of corneal endothelial cells, which are essential for maintaining corneal clarity through regulation of fluid balance [6,7,8,9]. Clinically, CMV corneal endotheliitis typically presents with characteristic keratic precipitates (KPs) of linear or coin-shaped morphology, accompanied by anterior chamber inflammation and recurrent episodes of elevated intraocular pressure (IOP) [3,6]. 

A critical complication of CMV corneal endotheliitis is secondary glaucoma, which is driven by direct viral cytopathic effects, host immune-mediated and inflammatory mechanisms leading to aqueous outflow dysfunction, sustained IOP elevation, and potential irreversible optic nerve damage [10,11,12,13,14]. This condition is frequently misdiagnosed or underrecognized, posing a considerable diagnostic and therapeutic challenge for ophthalmologists worldwide [15,16]. The insidious nature of CMV infection, together with its recurrence and progressive tissue damage, underscores the importance of early and accurate diagnosis followed by timely and appropriate therapeutic intervention to prevent irreversible corneal decompensation and glaucomatous optic neuropathy [17,18]. Unlike primary glaucoma, secondary glaucoma may be preventable if the underlying cause is identified and treated at an early stage. In contrast, delayed diagnosis or inadequate therapy can result in progressive glaucomatous optic neuropathy and, ultimately, irreversible vision loss [19]. 

Therefore, this review not only summarizes CMV corneal endotheliitis and its associated secondary glaucoma, but also specifically focuses on the clinical and mechanistic continuum linking endothelial infection to progressive outflow dysfunction and glaucomatous damage. Unlike prior broad reviews on CMV anterior segment infection, this review emphasizes unresolved diagnostic challenges, heterogeneity across published cohorts, limitations of current therapeutic evidence, and the pathogenic mechanisms underlying progression to secondary glaucoma.

## 2. Clinical Features

CMV infection of the anterior segment presents with a spectrum of clinical features that are often subtle but clinically meaningful. Clinical manifestations range from mild anterior segment inflammation to severe endothelial dysfunction and vision-threatening secondary glaucoma. Recognizing these features is essential for distinguishing CMV-related disease from other inflammatory or infectious conditions affecting the anterior segment. Key clinical features reported across representative studies are summarized in Table 1.

### 2.1. Clinical Features of CMV Infection

CMV infection is highly prevalent worldwide, with seroprevalence rates varying across populations, often reaching 60–90% in adults globally and up to 80% in certain regions [20,21]. In 2006, Koizumi et al. first identified CMV DNA in the aqueous humor of a patient with corneal endotheliitis, providing direct evidence of CMV involvement in anterior segment disease [22]. 

In immunocompetent individuals, CMV infection is usually asymptomatic, whereas in immunocompromised patients—such as transplant recipients, individuals with HIV/AIDS, patients with hematologic malignancies, or those receiving cancer chemotherapy—it may cause substantial morbidity and mortality [23,24,25,26,27,28,29,30,31]. However, accumulating evidence has established CMV as an important cause of anterior segment disease, including corneal endotheliitis, even in immunocompetent hosts. This condition predominantly affects middle-aged to elderly men, and the majority of reported cases originate from East Asia, particularly China and Japan [5,6,32,33,34,35,36,37]. This broader clinical context is also relevant to other virus-associated uveitic entities in East Asia, where ophthalmic presentation may provide an important clue to adult-acquired systemic viral infection [38,39,40].

### 2.2. Clinical Features of CMV Corneal Endotheliitis

CMV corneal endotheliitis exhibits a set of recognizable clinical features that can aid diagnosis when carefully assessed. These features are primarily observed through clinical examination and imaging modalities and are indicative of endothelial dysfunction and inflammation (Table 1).

#### 2.2.1. KPs

**Figure 1:**
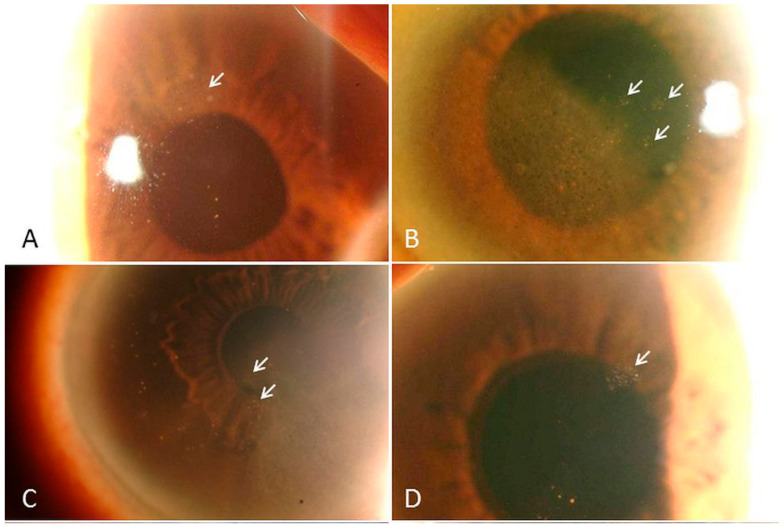
Slit-lamp photographs of patients with CMV corneal endotheliitis depict typical CMV endotheliitis, showing characteristic coin-shaped lesions (white arrows). These coin-shaped lesions are a characteristic and diagnostically suggestive finding in CMV corneal endotheliitis and are commonly observed in representative clinical cases (**A**–**D**). (adapted with permission from [34] 2015, Koizumi et al.).

KPs, representing inflammatory cell deposits on the corneal endothelium, are a consistent finding in CMV corneal endotheliitis and have been reported in nearly all cases in some series [41]. They can manifest in distinct morphological patterns that provide important diagnostic clues (Figure 1). Coin-shaped KPs are particularly suggestive of CMV infection and typically appear as round, relatively large deposits that are often pigmented. They may be diffusely distributed or confined to localized areas of the endothelium [6,34,42,43].

Linear KPs have also been noted in a substantial proportion of cases, sometimes associated with localized stromal edema [22,34,36,44,45]. Pigmented KPs are especially common in patients who develop CMV endotheliitis following endothelial keratoplasty, such as Descemet Membrane Endothelial Keratoplasty (DMEK) or Descemet Stripping Endothelial Keratoplasty (DSAEK) [15,46]. Taken together, the presence and pattern of KPs represent important clinical indicators of CMV corneal endotheliitis.

#### 2.2.2. Corneal Edema

Corneal edema results from endothelial barrier dysfunction and impaired pump activity, ultimately leading to corneal decompensation and reduced visual acuity [9,32,34]. It is a prominent clinical feature of CMV corneal endotheliitis and is observed in the majority of patients [6,8,35]. In a large Japanese case series involving 106 patients, corneal edema was present in 73.4% of eyes at the time of diagnosis [34]. 

The distribution of edema may be localized, presenting as disciform or sectoral involvement, or diffuse in more advanced or chronic cases, sometimes progressing to generalized bullous keratopathy [6,36,47]. Improvement or resolution of corneal edema is commonly used as a clinical marker of treatment response [41]. Conversely, persistent edema is associated with poorer visual outcomes and may necessitate surgical intervention, particularly when involving more than 75% of the corneal surface prior to treatment [48]. Corneal edema thus reflects the severity of endothelial damage and may coexist with trabecular dysfunction, contributing to ocular hypertension and secondary glaucoma. 

#### 2.2.3. Mild Anterior Chamber Inflammation

CMV corneal endotheliitis generally shows milder inflammatory signs than other forms of viral uveitis; however, anterior chamber inflammation is almost invariably present [32]. In the majority of cases, it appears as low-grade anterior chamber cells and flare. Notably, the degree of inflammation is often modest when compared with the extent of endothelial dysfunction or the level of IOP elevation [6,15]. The subtle inflammatory profile of CMV infection may obscure the viral etiology, leading to the progression of secondary disease [35]. 

#### 2.2.4. IOP Elevation

Elevated IOP is a hallmark feature of CMV anterior segment infection and a major contributor to the development of secondary glaucoma [8,11]. Patients frequently experience intermittent IOP spikes during periods of active inflammation [49]. These hypertensive episodes may be associated with blurred vision, ocular discomfort, mild ocular pain, photophobia, and conjunctival hyperemia.

Persistent or recurrent IOP elevation is of clinical concern, as it can lead to irreversible glaucomatous optic neuropathy if not promptly and adequately controlled [11,50]. Clinical studies have demonstrated that a substantial proportion of patients with CMV corneal endotheliitis ultimately develop glaucoma, with many requiring surgical interventions for IOP control [11,33]. Early diagnosis and timely initiation of antiviral and IOP-lowering therapy are therefore critical to reducing the risk of advanced glaucoma and the need for surgical management [18].

### 2.3. Clinical Features of Secondary Glaucoma

With progression to secondary glaucoma, IOP elevation often becomes persistent or frequently recurrent, representing a severe complication that can culminate in progressive visual deterioration [51]. Clinically, secondary glaucoma associated with CMV corneal endotheliitis exhibits a broad spectrum of manifestations, ranging from asymptomatic IOP elevation in early stages to advanced disease characterized by optic disk cupping and reproducible visual field loss [11]. 

Beyond these manifestations, acute hypertensive episodes may cause ocular pain, redness, and blurred vision, whereas chronic IOP elevation is often clinically silent until substantial optic nerve damage has occurred. Functional impairment typically manifests as progressive visual field defects, including scotoma enlargement and peripheral field constriction, with possible central vision involvement in advanced stages, reflecting irreversible glaucomatous optic neuropathy. Sustained or recurrent IOP elevation is also associated with characteristic structural changes, including optic nerve head remodeling and thinning of the retinal nerve fiber layer (RNFL) [49]. Mori et al. reported that 52.9% of patients with CMV corneal endotheliitis complicated by glaucoma exhibited moderate-to-advanced visual field defects, highlighting the severe impact on visual function [11]. 

The mechanisms underlying secondary glaucoma in CMV corneal endotheliitis are likely multifactorial and remain incompletely defined. Current evidence suggests that inflammatory responses and direct viral involvement of the TM synergistically impair aqueous humor outflow, leading to sustained IOP elevation [12,13,52]. Consequently, CMV-associated secondary glaucoma is often difficult to control, frequently requiring multiple IOP-lowering medications and, in a substantial proportion of cases, surgical intervention [11,50,53]. In a representative case series, Mori and colleagues reported secondary glaucoma in 32 of 34 eyes (94.1%) with CMV corneal endotheliitis; despite antiviral-centered therapy including topical 0.5% ganciclovir, approximately half of the patients ultimately required glaucoma surgery [11]. These observations highlight the need for early recognition, close monitoring of IOP, and timely intervention to preserve visual function in affected individuals [49].

## 3. Diagnosis

Accurate and timely diagnosis of CMV corneal endotheliitis and associated secondary glaucoma is essential for effective management and prevention of irreversible vision loss. Given the often subtle and non-specific presentation, diagnosis typically requires integration of characteristic clinical findings with laboratory confirmation. Additionally, distinguishing CMV corneal endotheliitis from other conditions is crucial, as misdiagnosis may lead to the malignant progression of the disease.

### 3.1. Clinical Tests

Clinical examinations form the basis of both initial evaluation and longitudinal follow-up in patients with CMV corneal endotheliitis and secondary glaucoma. They guide diagnostic suspicion and allow assessment of disease activity, progression, and response to treatment.

#### 3.1.1. Slit-Lamp Biomicroscopy

**Figure 2:**
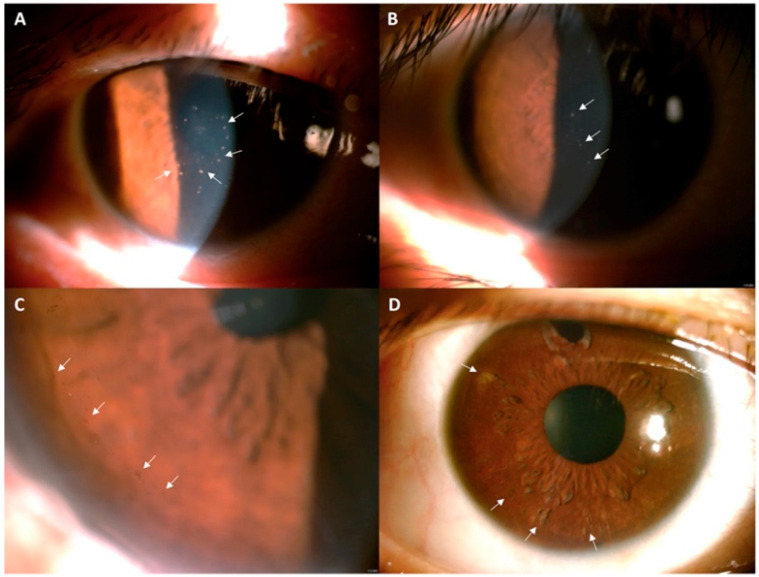
Slit-lamp images showing different manifestations of CMV corneal endotheliitis in various cases. Central corneal coin-shaped KPs (white arrows) (**A**), mutton-fat KPs (white arrows) (**B**), peripheral corneal pigmented KPs (white arrows) (**C**), and iris atrophy with a moth-eaten appearance (white arrows) (**D**). These images illustrate the clinical features used for diagnosing CMV corneal endotheliitis in patients at Cathay General Hospital in Taipei, Taiwan. (adapted with permission from [41] 2022, Kuo et al.).

Slit-lamp biomicroscopy allows for a detailed assessment of the cornea, anterior chamber, and iris, and is essential for identifying potential characteristic signs of CMV corneal endotheliitis; however, caution should be exercised when interpreting certain imaging findings, as their specificity may vary (Figure 2). Key findings include corneal edema (localized, disciform, or diffuse) and KPs, particularly coin-shaped KPs, as well as linear or diffusely distributed pigmented KPs [6,8,34,42]. Mild anterior chamber inflammation (cells and flare) and iris abnormalities such as “moth-eaten” iris atrophy may also be observed [6,32,41]. 

Combined with Goldmann applanation tonometry, slit-lamp examination facilitates IOP assessment, which is frequently elevated in CMV anterior segment infection and is central to diagnosing and monitoring secondary glaucoma [11]. Given the dynamic nature of these signs and their evolution with therapy, repeated slit-lamp examinations are essential for monitoring treatment response [54].

#### 3.1.2. AS-OCT 

Anterior segment optical coherence tomography (AS-OCT) is a non-invasive imaging modality that provides high-resolution cross-sectional visualization of anterior segment structures. In CMV corneal endotheliitis, AS-OCT may reveal hyperreflective protrusions on the posterior corneal surface with variable morphologies, including dendritic, dome-shaped, quadrangular, or saw-tooth patterns [55]. These findings are thought to correspond to inflammatory deposits and/or structurally altered endothelial cells. Importantly, reduction or resolution of posterior corneal hyperreflectivity following antiviral therapy highlights the potential utility of AS-OCT for both diagnosis and longitudinal monitoring of treatment response [55].

#### 3.1.3. Specular Microscopy

Specular microscopy is a key tool for evaluating the corneal endothelium, providing quantitative and qualitative assessment of endothelial cell density (ECD), morphology, and polymegethism/pleomorphism. In CMV corneal endotheliitis, reduced ECD is commonly observed and reflects virus-associated endothelial injury [18,49]. 

A particularly distinctive finding is the presence of enlarged endothelial cells containing viral inclusion bodies, commonly referred to as “owl’s eye” cells, which are characteristic manifestations often considered suggestive of CMV infection [16,56]. Supporting this observation, recent in vitro studies have demonstrated markedly enlarged endothelial cells with a hyperreflective nucleus surrounded by a hyporeflective halo, corresponding to the “owl’s eye” appearance seen clinically [3,16]. The emergence and subsequent resolution of these cells during treatment may provide a non-invasive means of assessing disease activity and therapeutic response [16,54].

#### 3.1.4. IVCM

In vivo confocal microscopy (IVCM) is a non-invasive, high-resolution imaging modality that enables cellular-level visualization of the corneal endothelium and associated inflammatory changes [57,58]. Its ability to track microstructural changes over time makes it useful for supporting diagnosis and monitoring treatment response, particularly through longitudinal assessment of endothelial cells and KPs [58]. 

IVCM may also aid etiologic differentiation in viral endotheliitis. Comparative analyses have reported distinct endothelial patterns among herpesviruses, and CMV endotheliitis is notably associated with “owl’s eye”-like morphology [59,60]. 

#### 3.1.5. Assessment for Secondary Glaucoma

Regular assessment for secondary glaucoma is integral to preventing irreversible glaucomatous damage in CMV corneal endotheliitis. This involves serial IOP measurements, gonioscopy, and structural and functional evaluation of the optic nerve. Elevated IOP is a frequent complication, and its control is critical to prevent glaucomatous optic neuropathy [11]. Gonioscopy may evaluate the anterior chamber angle and reveal signs of inflammation in the angle or peripheral anterior synechiae, which can contribute to IOP elevation [18]. 

Optic disk assessment for progressive cupping and RNFL thickness measurements, typically obtained by optical coherence tomography (OCT), are essential for detecting and monitoring glaucomatous damage. However, fundus evaluation may be limited in eyes with marked corneal edema, necessitating repeat posterior-segment assessment after corneal clarity improves [61]. Objective morphologic evidence of glaucomatous progression, including RNFL thinning, further highlights the need for standardized structural monitoring to guide therapy [49].

### 3.2. Laboratory Tests

Laboratory tests on aqueous humor samples are indispensable for confirming the diagnosis of CMV anterior segment infection and guiding treatment. Among the available modalities, emerging diagnostic techniques are becoming more effective in establishing viral etiology.

#### 3.2.1. Aqueous Humor PCR and qPCR

Polymerase chain reaction (PCR) and quantitative PCR (qPCR) are the gold standard for diagnosing active CMV infection in the anterior segment and for differentiating CMV from other herpesviruses such as HSV and VZV that have similar clinical presentations with CMV corneal endotheliitis [22,32,62,63,64,65,66]. 

Higher CMV DNA copy numbers in aqueous humor have been associated with active inflammation, more frequent recurrences, and IOP elevation [67]. Multicenter studies have reported high diagnostic performance of qPCR for ocular CMV infection, with sensitivities of approximately 90% [67]. Beyond diagnosis, qPCR may provide value for monitoring treatment response, as declining viral loads are consistent with effective antiviral suppression. Conversely, persistently elevated or rising CMV DNA levels may suggest inadequate viral control, increased risk of recurrence, or unfavorable surgical outcomes [18,68].

Nevertheless, aqueous humor PCR has inherent limitations. Anterior chamber paracentesis is an invasive procedure that carries risks such as IOP spikes, wound leaks, and potential infection or hemorrhage [69]. In addition, CMV DNA may not be detectable during the initial visit, and multiple tests may be required for a conclusive diagnosis, particularly in cases with less severe inflammation or those undergoing antiviral treatment [64]. Furthermore, false-negative results can also occur, particularly in settings of low viral load, localized endothelial infection, or sampling outside the active phase. In refractory cases, CMV DNA has been detected in surgically excised Descemet’s membrane and corneal endothelium despite negative aqueous PCR, highlighting the potential role of tissue analysis when clinical suspicion remains high [45]. In addition, while PCR confirms the presence of CMV, it may not always differentiate between active infection and latent viral presence, especially with low copy numbers. However, a standardized threshold for CMV DNA load in aqueous humor for clinical diagnosis may not have been clearly established [70]. Additionally, the variation in CMV DNA copy numbers across institutions, sometimes differing by up to fourfold for the same sample, can likely be attributed to differences in primers, reagents, equipment, and target amplification protocols, as well as variations in DNA standards [64,68].

#### 3.2.2. IgG and IgM Antibodies

Serological testing for CMV-specific IgG and IgM antibodies can provide information about past or recent systemic CMV exposure. CMV IgG positivity reflects prior infection and latent viral carriage, which is common in the general population [53,61], and, therefore, does not establish active intraocular infection. CMV IgM antibodies typically indicate recent or primary systemic infection; however, ocular CMV disease in immunocompetent individuals often represents localized viral reactivation without systemic viremia or a detectable IgM response [22].

As a result, while serologic testing may offer supportive contextual information, it is generally insufficient for confirming active CMV anterior segment infection. Detection of CMV DNA in aqueous humor by PCR remains the principal diagnostic approach in this setting.

#### 3.2.3. Emerging Diagnostic Technologies

Beyond these, emerging diagnostic technologies are being explored. Metagenomic Next-Generation Sequencing (mNGS) of aqueous humor has shown promise as a comprehensive diagnostic tool, particularly in cases where routine qPCR may yield negative or inconclusive results, or when differentiating between multiple potential viral etiologies is challenging [71]. In one comparative study of patients with suspected CMV endotheliitis, qPCR detected CMV in 36.4% of cases, whereas mNGS identified CMV in all confirmed cases, suggesting a potentially higher diagnostic yield and the added advantage of detecting alternative or co-infecting pathogens [71]. Nevertheless, clinical laboratory operations for mNGS, in the absence of full automation, may require significant hands-on time and highly trained personnel. Repetitive tasks, such as pipetting, increase the risk of procedural errors. While mature automation of sample handling with robotic systems can improve efficiency, it also significantly increases costs. Additionally, due to high reagent expenses, manual library preparation, bioinformatics analysis, and the need for regulatory oversight, mNGS remains costly and is associated with longer overall turnaround times. Together, these factors limit its widespread clinical adoption, and future technological advancements are necessary to overcome these challenges [71,72]. 

### 3.3. Differential Diagnosis

Differentiating CMV corneal endotheliitis from Posner–Schlossman syndrome (PSS) remains clinically challenging due to overlapping features. A study involving 18 patients with CMV corneal endotheliitis found that nearly half of the cases were initially diagnosed as PSS [11]. CMV corneal endotheliitis typically presents with coin-shaped or linear KPs and is commonly associated with progressive severe corneal edema. As the corneal edema worsens, IOP generally increases gradually. The disease tends to follow a chronic or recurrent course. In contrast, PSS often presents with medium-sized, mutton-fat-like KPs, less frequent corneal edema, and recurrent acute episodes of markedly elevated IOP, often reaching 40–70 mmHg during an attack [73]. Recent studies have shown that a significant subset of PSS patients also test positive for CMV. A meta-analysis of 21 studies found that approximately 44.5% of PSS patients were CMV-positive, with these eyes exhibiting lower corneal endothelial cell density [33].

Furthermore, CMV endotheliitis is associated with secondary glaucoma, which typically manifests in the mid-to-late stages of disease progression, often after irreversible damage has occurred. In contrast, chronic hypertensive anterior uveitis is characterized by persistent intraocular inflammation lasting for at least 3 months. Research has shown that among CMV-positive patients with hypertensive anterior uveitis, 66.7% required glaucoma surgery despite treatment, due to ongoing uncontrolled IOP elevation [74].

These findings suggest that some other anterior ocular diseases may indeed be closely associated with CMV infection. However, the distinction between them is currently primarily based on clinical symptom differences, CMV DNA detection, response to antiviral treatment, and anti-glaucoma therapies. The exact relationship between them, including whether CMV endotheliitis is a primary etiological factor, a coexisting infection, or a secondary complication, remains unclear and warrants further investigation.

## 4. Treatment

Management of CMV corneal endotheliitis complicated by secondary glaucoma requires integrated control of viral activity, intraocular inflammation, and IOP. Antiviral therapy remains the cornerstone of treatment. Anti-inflammatory agents and IOP-lowering strategies are essential adjuncts. Surgical intervention is reserved for cases refractory to medical therapy or complicated by corneal decompensation or uncontrolled glaucoma (Table S1).

### 4.1. Medical Therapy

#### 4.1.1. Antiviral

Antiviral therapy is fundamental in CMV corneal endotheliitis, aiming to suppress viral replication, mitigate inflammation, and prevent further endothelial injury and IOP elevation. Ganciclovir and valganciclovir are the preferred first-line agents.

##### Topical Ganciclovir

Topical ganciclovir, typically in 0.15% or 2.0% formulations, is widely used and offers a local approach with fewer side effects. The efficacy of 0.15% ganciclovir gel has been proved to be safe and effective for CMV corneal endotheliitis patients [75]. In the study by Koizumi et al., intensive dosing (six times daily for 12 weeks) was associated with reduced aqueous CMV DNA copy numbers, and six of seven eyes achieved corneal clarity with preserved endothelial function [68]. 

However, concerns have been raised regarding low intraocular drug penetration and suboptimal efficacy with the 0.15% gel formulation [76,77]. To address this limitation, higher-concentration formulations have been investigated. Topical 2.0% ganciclovir achieves markedly higher aqueous humor concentrations (approximately 1200 ng/mL), exceeding the inhibitory dose for CMV replication, and results in higher rates of undetectable CMV titers compared with the 0.15% gel (approximately 17 ng/mL) [17,76]. Long-term use of 2.0% ganciclovir has also been associated with reduced KPs, improved IOP control, and lower recurrence rates [78]. Nevertheless, 2.0% ganciclovir drops are not commercially available in all regions, and adherence may be challenging due to frequent dosing requirements [77]. 

##### Systemic Valganciclovir

Oral valganciclovir is often used for severe cases, bilateral involvement, or when topical therapy alone is insufficient [42,44,53]. Some studies suggest that continuous low-dose oral valganciclovir may be effective as maintenance therapy to prevent recurrences [44]. In addition, intravitreal injections of ganciclovir are an option for severe or refractory cases, or when rapid intraocular drug levels are required. This approach, often combined with oral valganciclovir, has shown a reduction in long-term recurrence and improvement in corneal endothelial cell counts and visual acuity [79]. 

However, systemic therapy is costly and carries a higher risk of adverse effects, including myelosuppression, neutropenia, and thrombocytopenia [80]. In addition, systemic antiviral therapy may be associated with higher recurrence rates. A retrospective clinical study of oral valganciclovir for CMV anterior segment inflammation, including endotheliitis, found that while oral valganciclovir was effective in controlling inflammation, 38.5% of treated eyes experienced recurrence after treatment cessation and required retreatment [81]. Additionally, another large cohort study noted that approximately 72% of patients still experienced recurrence after completing antiviral treatment with oral valganciclovir followed by topical therapy [81]. Furthermore, in solid organ transplant recipients undergoing CMV prophylaxis, valganciclovir can also be associated with toxicity and breakthrough CMV DNAemia [82].

Although different treatment approaches offer advantages, such as improved clinical outcomes or fewer side effects, the existing evidence is largely derived from retrospective cohorts and case series. Currently, there is a lack of prospective randomized controlled trials directly comparing various topical concentrations (e.g., 0.15% vs. 2%) or treatment regimens (e.g., topical vs. systemic) in CMV endotheliitis, and further investigation is needed. Therefore, the selection of topical therapy, systemic therapy, or a combination thereof should be based on a comprehensive assessment of disease severity, patient tolerance, treatment response, and the presence of secondary complications, including glaucoma, and other factors [35,83,84,85].

#### 4.1.2. Anti-Inflammatory

Topical corticosteroids are frequently used in combination with antivirals to control inflammation in CMV corneal endotheliitis. In clinical practice, treatment often combines antiviral therapy with topical corticosteroids (1% prednisolone acetate eye drops) to reduce corneal edema and anterior chamber inflammation [56]. In some cases, systemic corticosteroids may be used for severe inflammation. 

Nevertheless, corticosteroid use requires caution [86,87,88,89]. Steroids may induce reactivation of latent CMV infection and exacerbation of viral replication, especially in cases of underlying viral etiology [53,90]. In addition, prolonged corticosteroid exposure may induce cytoskeletal remodeling of TM cells, thereby contributing to steroid-induced glaucoma [91]. Optimal management, therefore, requires minimizing steroid exposure while ensuring adequate viral suppression and close monitoring for IOP elevation [18]. 

#### 4.1.3. IOP Control

Control of CMV-associated IOP elevation is critical to prevent secondary glaucoma and preserve visual function [92]. First-line management typically includes topical anti-glaucoma medications such as prostaglandin analogs, β-blockers, α-agonists, and carbonic anhydrase inhibitors [11,93,94,95]. However, IOP elevation in CMV anterior segment infection can be difficult to control, often requiring combination therapy and frequent monitoring [18,96]. 

Rho kinase (ROCK) inhibitors have recently attracted interest as adjunctive therapy. Experimental data suggest that ROCK inhibition may support corneal endothelial function and reduce apoptosis while also lowering IOP [97,98]. Although these findings are promising, clinical evidence remains limited, and further studies are required to define their role in CMV-associated disease. Despite maximal medical therapy, a substantial proportion of patients ultimately require surgical intervention for refractory IOP elevation.

### 4.2. Surgical Therapy

#### 4.2.1. Corneal Transplantation

Advanced CMV corneal endotheliitis may progress to irreversible endothelial failure and bullous keratopathy, necessitating corneal transplantation. Endothelial keratoplasty, including Descemet’s stripping automated endothelial DSAEK and DMEK, has become the preferred surgical approach, providing faster visual recovery and fewer complications than penetrating keratoplasty [90,99].

However, CMV infection poses specific challenges in the keratoplasty setting. CMV corneal endotheliitis can occur after endothelial transplantation and frequently mimics immunologic graft rejection, potentially leading to graft failure if mismanaged [42,100,101,102,103]. Accordingly, preoperative aqueous humor PCR testing for CMV is strongly recommended when clinical suspicion exists. In CMV-positive patients, perioperative and prolonged postoperative antiviral therapy is often required to reduce recurrence risk and improve graft survival [104,105,106,107,108]. Despite prophylaxis, recurrence remains clinically relevant, underscoring the need for long-term postoperative surveillance [104].

#### 4.2.2. Glaucoma Surgery

In refractory CMV-associated secondary glaucoma, surgical IOP-lowering procedures may be required. Trabeculectomy remains a commonly performed filtration surgery and has been reported in a substantial proportion of patients with CMV corneal endotheliitis complicated by glaucoma [11,49,53]. However, trabeculectomy carries risks, including accelerated corneal endothelial cell loss and bullous keratopathy, particularly in eyes with pre-existing endothelial compromise [109]. 

Glaucoma drainage devices (GDDs) are often considered in eyes with extensive conjunctival scarring, prior failed filtration surgery, or when trabeculectomy is less likely to succeed. However, they may carry the risk of intraocular infection and device exposure. A meta-analysis of 38 studies involving 3255 eyes found that the incidence of exposure to GDDs ranged from 0% to 12%. Additionally, a nine-year retrospective study reported an endophthalmitis rate of 1.7% following the implantation of Ahmed drainage devices in 542 eyes [7]. 

Minimally invasive glaucoma surgery (MIGS) has emerged as a safer alternative to traditional incisional glaucoma surgery, particularly for patients with mild to moderate disease. MIGS procedures are designed to minimize tissue trauma, reduce recovery time, and lower the risk of serious complications relative to trabeculectomy and glaucoma drainage implants, mainly through ab interno approaches and conjunctival sparing techniques. However, although short-term outcomes show promising reductions in intraocular pressure and medication burden, long-term efficacy data are limited, and comparative evidence against other approaches remains to be further explored [50,110,111].

In addition, MicroPulse transscleral laser therapy (MP-TLT) has been investigated as an alternative, non-invasive approach for refractory glaucoma, potentially reducing IOP via enhanced uveoscleral outflow [112]. Unlike conventional surgical procedures, it is a laser treatment targeting the uveoscleral outflow pathway to reduce IOP. MP-TLT is often considered for cases of refractory glaucoma or in patients who are at high risk for more invasive surgery. This treatment is associated with a lower risk of infection and surgical failure and is repeatable. However, it is less effective than traditional surgical treatments in achieving the target IOP levels, and its long-term efficacy requires further investigation [113,114]. Surgical choice should be individualized based on the extent of endothelial damage, inflammatory activity, angle status, and surgical history.

Although some clinical articles have outlined current treatment regimens, there is still no consensus or standardized guidelines regarding specific medication timing, dosage, and administration protocols. Furthermore, in cases of worsening corneal pathology or secondary glaucoma, surgical intervention may be necessary. However, the indications for surgery remain unclear, and there is a notable absence of standardized surgical guidelines.

### 4.3. Long-Term Management and Follow-Up

CMV corneal endotheliitis is a chronic and relapsing condition. Long-term management and rigorous follow-up are therefore critical. Continuous low-dose antiviral maintenance therapy, particularly with topical ganciclovir, has been shown to reduce relapse frequency significantly [76,78]. For instance, topical 2.0% ganciclovir solution can reduce recurrence rates to as low as 0.13 relapses per year [78]. Low-dose oral valganciclovir may also be effective in selected patients [44].

Regular follow-up examinations should include slit-lamp biomicroscopy to monitor for KPs, corneal edema, and anterior chamber inflammation; IOP measurements to detect and manage ocular hypertension; specular microscopy to assess ECD and morphology, including the presence of owl’s eye cells; and visual field testing and OCT of the optic nerve head and RNFL to monitor for glaucomatous progression [16,49]. The duration of antiviral treatment, especially systemic therapy, should be individualized based on clinical response, viral load, and the patient’s immune status, balancing efficacy with potential side effects [90,115]. Early diagnosis and adherence to a standardized follow-up protocol are paramount to reducing recurrence rates, preventing glaucomatous damage, and ultimately improving both visual acuity and IOP outcomes [8,18].

## 5. Risk Factors

Identifying risk factors for CMV corneal endotheliitis and its progression to secondary glaucoma is crucial for early detection and targeted preventive strategies. Multiple factors have been associated with disease development and severity.

### 5.1. Age and Gender

A prominent demographic pattern in CMV corneal endotheliitis and subsequent secondary glaucoma is its predominance in middle-aged to elderly men [36,74]. 

Multiple studies, particularly from East Asia, report a marked male predominance and a mean age typically in the sixth to seventh decade. In the Japan Corneal Endotheliitis Study of 106 patients, the mean age was 66.9 years, with 85 males and 21 females [34]. Similarly, a study focusing on glaucoma associated with CMV corneal endotheliitis reported a mean age of 69.1 years, with 31 of 34 patients being male [11]. Furthermore, the data presented in Table 1 are highly relevant to the epidemiological trends discussed.

This demographic distribution suggests that age-related immune senescence and hormonal influences may contribute to CMV reactivation and ocular involvement in this group. Consistently, a recent study examining predictors of CMV corneal endotheliitis after corneal transplantation identified male sex as a significant risk factor [116]. Moreover, PSS, which are frequently linked to CMV infection and secondary glaucoma, also tend to occur in similar demographic groups [33]. The mechanisms underlying this predilection are not fully understood but may involve differences in immune responses or exposure patterns.

### 5.2. Host Immune Status

Immunosuppression, such as immunosuppressive medications, can also influence the risk of CMV reactivation and CMV-related anterior segment disease. 

In a reported case series, irreversible ocular damage was observed in three patients receiving long-term topical tacrolimus and corticosteroid therapy, which may have promoted reactivation of latent CMV and precipitated CMV corneal endotheliitis. Although topical 0.5% ganciclovir improved endothelial inflammation, final outcomes remained poor so all patients required cataract surgery, and two required glaucoma surgery [90]. Patients undergoing keratoplasty who require systemic immunosuppressants are also at increased risk of CMV reactivation and subsequent endotheliitis [42,104]. More broadly, CMV remains a major concern in immunocompromised hosts, such as solid organ and hematopoietic stem cell transplant recipients, in whom CMV can cause severe systemic and ocular complications [23,24,25,117]. Furthermore, immune reconstitution inflammatory syndrome (IRIS) occurs in HIV-infected individuals shortly after the initiation of antiretroviral therapy, triggering the reactivation of latent CMV infections. This reactivation can lead to tissue-destructive inflammation and potentially severe complications, including the coinfection of tuberculosis in ocular tissues [118,119]. These observations underscore the importance of vigilant monitoring and proactive antiviral strategies in immunosuppressed patients.

### 5.3. Prolonged and Recurrent CMV Infection

A prolonged disease course, misdiagnosis or delayed recognition, inadequate viral suppression, and frequent recurrences have been associated with increased risk of corneal endothelial damage and progression to secondary glaucoma [120,121,122]. 

Studies suggest that higher aqueous CMV DNA levels and longer disease duration correlate with more substantial endothelial cell loss [120,123]. Furthermore, frequent recurrences are often observed when antiviral treatment is discontinued or inadequate, with reported relapse rates reaching 60–70% after cessation in some series, underscoring the need for individualized maintenance strategies in selected patients [76]. In addition, misdiagnosis or delayed recognition, often due to clinical manifestations overlapping with other uveitic entities or graft rejection, may lead to inappropriate corticosteroid escalation without adequate antiviral coverage, thereby exacerbating viral activity and accelerating ocular damage, including secondary glaucoma [101]. Even low-level persistent viral activity may contribute to cumulative endothelial injury, increasing the likelihood of corneal decompensation and repeat graft failure after keratoplasty [124].

### 5.4. Pre-Existing Ocular Conditions

Pre-existing ocular diseases and prior intraocular surgery appear to predispose eyes to CMV corneal endotheliitis and secondary glaucoma [125,126]. In a case series of CMV corneal endotheliitis complicated by glaucoma (n = 34), 94.1% of eyes had a pre-existing diagnosis of glaucoma and had been treated for a mean of 10.0 ± 10.1 years before CMV endotheliitis was definitively diagnosed. Additionally, 47.1% had glaucoma in the fellow eye and 38.2% had a history of PSS, suggesting a predisposition to hypertensive anterior segment inflammation and limited optic nerve reserve [11]. 

Despite IOP reduction with medical therapy (mean 22.4 ± 10.6 mmHg to 14.9 ± 7.9 mmHg, *p* < 0.01), 47.1% of eyes still required glaucoma surgery during follow-up, indicating that cumulative trabecular damage can render IOP increasingly difficult to control [11]. Furthermore, multivariable analyses have identified that a previous history of anterior uveitis (OR 25.31, 95% CI 8.22–95.19) and a previous history of glaucoma (OR 6.25, 95% CI 1.17–115.90) were each significantly associated with postoperative CMV corneal endotheliitis [116].

In addition, long-term cohort studies of PCR-confirmed CMV anterior segment infection have shown that prior corneal transplantation before CMV diagnosis is an independent predictor of molecular recurrence (hazard ratio 6.81, 95% CI 1.21–38.23, *p* = 0.029) [79,127]. Surgical disruption of the ocular immune environment, combined with recurrent viral activity, may accelerate endothelial loss and chronic anterior segment dysfunction, thereby increasing the risk of secondary glaucoma.

### 5.5. Other Risk Factors

Metabolic disorders such as diabetes mellitus (DM), hyperlipidemia, and hypertension have been associated with CMV infection and the progression of CMV-related secondary glaucoma. 

Research indicates that individuals with DM experience a significantly higher incidence of endotheliitis compared to those without diabetes. This difference may be attributed to abnormalities in cell-mediated immunity due to hyperglycemia, as well as the impairment of the blood–aqueous barrier, which may facilitate the infiltration of virus-infected cells into the corneal endothelium [125,128]. Additionally, diabetes can lead to retinal ischemia, triggering the release of vascular endothelial growth factor (VEGF), which promotes neovascularization within the anterior chamber angle. This process obstructs trabecular outflow, resulting in increased IOP and ultimately contributing to the development of secondary glaucoma [129].

Hypertension, on the other hand, may increase the risk of CMV infection in the eye by inducing microvascular damage, which impairs the eye’s immune defenses [130]. Additionally, high blood pressure may exacerbate ocular blood flow insufficiency, accelerating CMV-induced corneal endothelial cell damage [131]. Elevated intraocular pressure, a common consequence of hypertension, further worsens disease progression [132].

Dyslipidemia can suppress immune responses, which may allow CMV to persist within the eye without being effectively cleared, thereby promoting continuous ocular inflammation [133]. Moreover, the combined effects of oxidative stress and inflammation can disrupt TM function, impair aqueous humor drainage and increase IOP, which exacerbates the development of secondary glaucoma [134,135].

Therefore, although many risk factors have already been identified as important contributors to the disease, further studies are needed to better understand the role of CMV in the development of ocular pathologies and secondary glaucoma, as well as to explore potential additional risk factors.

## 6. Pathogenesis

CMV corneal endotheliitis and its secondary glaucoma reflects a complex interplay among direct viral cytopathic effects, inflammatory mediators and host immune responses. These processes converge on progressive endothelial dysfunction, impaired aqueous humor outflow, and irreversible optic nerve damage. 

### 6.1. Pathogenesis of CMV Corneal Endotheliitis

Following primary infection, CMV establishes lifelong latency in multiple host tissues, including ocular tissues such as the corneal endothelium, ciliary body, and TM [136,137]. Within the eye, viral persistence may be facilitated by the immune-privileged anterior chamber environment, where anterior chamber-associated immune deviation (ACAID) occurs as viruses such as CMV exploit TGF-β to promote the generation of regulatory T cells and suppress the responses of CD4^+^ TH1 and TH2 cells [138]. This immune modulation creates a low-immune surveillance environment, allowing the virus to persist without being cleared [139,140,141,142]. 

Under conditions that disrupt host–virus equilibrium, such as systemic immunosuppression, local or systemic corticosteroid exposure, intraocular surgery, trauma, or other stressors, latent CMV may reactivate. Reactivation is characterized by immediate-early viral gene expression and induction of pro-inflammatory cytokines such as TNF-α, which can further enhance viral replication and sustain a self-amplifying inflammatory loop [95,97]. Experimental evidence further indicates that MEK-ERK signaling is critical for CMV reactivation and suggests that targeting this pathway may help limit viral reactivation and downstream pathogenesis [143]. In addition, studies have shown that the EGFR-AKT-PI3K signaling axis, along with related Rho and MAPK pathways, also plays a crucial role in reactivation [137]. Furthermore, recent research has found that viral factors such as UL8-mediated receptor degradation and miR-UL36’s post-transcriptional regulation further enhance viral reactivation by suppressing Notch signaling. These factors accomplish this by degrading Notch3 and downregulating the downstream transcriptional regulator RBPJ [144,145]. Despite these insights, the precise mechanisms underlying CMV’s dormancy and reactivation remain elusive. Moreover, repeated latency–reactivation cycles are thought to drive recurrent or chronic CMV corneal endotheliitis and provide a mechanistic basis for subsequent ocular hypertension and glaucomatous damage [85,146].

After reactivation, CMV corneal endotheliitis appears to involve both direct viral cytopathic injury and host immune inflammation [5,16]. Active viral replication within corneal endothelial cells disrupts cellular metabolism and ultrastructure, resulting in cellular swelling, cytoplasmic degeneration, and impaired endothelial function [7]. Viral antigen expression also provokes a cellular immune response. Lymphocytes, predominantly CD8^+^ T cells, together with monocytes accumulate at the endothelial surface and adhere to injured cells, further exacerbating tissue damage [120]. Cytokines released at the endothelial surface further amplify local inflammation and tissue injury [147].

Meanwhile, corneal endothelial cells are active participants in the immune response. Following CMV infection, they activate both innate and adaptive immune pathways. This response includes upregulation of type I interferon signaling, activation of pattern-recognition receptors, and secretion of chemokines such as CXCL10 [67]. These cells can also directly stimulate CD8^+^ T cell proliferation and interferon-γ secretion, indicating their role in activating acquired immunity [67].

Both direct viral injury and immune-mediated inflammation can culminate in endothelial pump failure. Experimental and clinical evidence suggests that viral proteins and the inflammatory microenvironment may induce mitochondrial dysfunction and reduce Na^+^/K^+^-ATPase activity, which is essential for maintaining corneal deturgescence. As pump capacity declines, aqueous fluid accumulates within the corneal stroma, producing the characteristic focal corneal edema observed in CMV endotheliitis. Progressive endothelial cell loss and persistent pump dysfunction, therefore, constitute the immediate basis for corneal decompensation and loss of transparency [5].

### 6.2. Pathogenesis of Secondary Glaucoma Induced by CMV Corneal Endotheliitis

Elevated IOP is a central feature of CMV corneal endotheliitis and a major contributor to secondary glaucoma. IOP elevation likely results from a combination of acute aqueous outflow obstruction and chronic TM damage [148].

As endothelial injury progresses, cellular debris, inflammatory cells, and viral particles are released into the anterior chamber [6,46,74,123,149]. These materials may accumulate within the TM, physically obstructing aqueous outflow and increasing outflow resistance. Pigmented inflammatory deposits may further exacerbate this process, contributing to acute IOP spikes [74]. Animal experiments have also shown that swelling of the trabecular lamellae and reduction in the intertrabecular spaces may block the outflow pathway. Over time, chronic inflammatory injury and debris accumulation may promote irreversible TM scarring and dysfunction, contributing to persistent secondary glaucoma [109]. 

In addition, CMV exhibits tropism for multiple ocular anterior segment tissues, and infection of TM cells has been proposed as a direct mechanism for outflow impairment [13,150]. In vitro studies have shown that CMV infection of human TM cells activates interferon signaling and upregulates inflammatory cytokines and chemokines, including IL-8 and CCL2 [13]. This infection-induced cellular activation can alter the dynamics of TM cells, leading to increased cell motility and contraction, which are dependent on CXCR2 and CCR2 signaling pathways, respectively [13]. Furthermore, emerging evidence suggests that HCMV-induced elevation in IL-8 triggers pathological remodeling of the TM actin cytoskeleton via the Rac1/Cdc42 GTPase pathways, reducing the effective pore size of the trabecular lamellae and acutely escalating aqueous humor outflow resistance [13]. Direct viral infection may also induce TM cell dysfunction, apoptosis, or necrosis, further compromising the structural integrity and filtering capacity of the outflow pathway [147,151]. In the chronic phase, persistent inflammation co-opts the TGF-β1/Smad signaling axis to drive excessive extracellular matrix deposition and TM stiffening. Recent studies also highlight viral-mediated suppression of TEAD1 transcriptional activity, which impairs the mechanical stress-sensing capabilities of TM cells, ultimately culminating in refractory secondary glaucoma [152].

Persistent or recurrent IOP elevation ultimately drives optic nerve damage. When IOP exceeds the compensatory capacity of the lamina cribrosa and its vascular support, axoplasmic transport in retinal ganglion cell axons becomes impaired, apoptotic loss accelerates, and glaucomatous optic neuropathy ensues. Clinically, this manifests as progressive optic disk cupping with neuroretinal rim thinning and corresponding visual field loss. Thus, recurrent CMV endotheliitis can initiate a pathogenic cascade from viral activity to outflow dysfunction and end-organ optic nerve damage, and the resulting secondary glaucoma is often difficult to control medically. These observations underscore the importance of early and sufficiently potent antiviral suppression, appropriately balanced anti-inflammatory therapy, and proactive IOP management to interrupt disease progression and prevent irreversible visual impairment [153].

In summary, the pathogenesis of CMV corneal endotheliitis and secondary glaucoma is multifactorial and remains incompletely understood. A deeper mechanistic understanding will be essential for developing improved diagnosis and therapies.

## 7. Conclusions

CMV infection of the anterior segment, predominantly manifesting as corneal endotheliitis, represents a clinically significant and often challenging ocular condition with a high propensity for progression to secondary glaucoma. This review has summarized key clinical features, diagnosis, therapeutic strategies, risk factors and putative pathogenic mechanisms.

The clinical hallmarks of CMV corneal endotheliitis, including coin-shaped or linear keratic precipitates, corneal edema, mild anterior chamber inflammation, and recurrent IOP spikes, provide important diagnostic clues [6,34]. However, the definitive diagnosis mainly relies on laboratory confirmation, with aqueous humor qPCR testing serving as the gold standard for detecting CMV DNA and quantifying viral load [11,34]. Adjunctive imaging modalities, including specular microscopy, anterior segment OCT, and in vivo confocal microscopy, offer valuable insights into endothelial pathology, assist in differentiating CMV endotheliitis from other conditions, and facilitate monitoring of treatment response [58,154]. 

Effective management necessitates a prompt and appropriate therapeutic approach. Antiviral therapy, primarily with topical ganciclovir or systemic valganciclovir, is essential to suppress viral replication and inflammation [44,78,83]. Equally important is timely IOP management. Although medical therapy may provide initial IOP control, a considerable proportion of patients ultimately require surgical intervention. Surgical planning must be cautious, given the elevated risks of endothelial cell loss, surgical failure, and postoperative corneal decompensation [149]. Because recurrence is common after treatment discontinuation, long-term maintenance antiviral therapy is often necessary to limit cumulative endothelial and optic nerve damage [44]. 

Several risk factors contribute to the development and severity of CMV-associated secondary glaucoma, including demographic predisposition, host immune status, prolonged and recurrent CMV infection and a history of pre-existing ocular disease or intraocular surgery [48]. These factors interact to promote viral reactivation, sustained inflammation, aqueous outflow dysfunction, and glaucomatous optic nerve injury [155].

Currently, a significant challenge is that randomized controlled trials evaluating treatment efficacy for CMV endotheliitis and its associated secondary glaucoma are still lacking. Consequently, standardized treatment guidelines for both conditions have yet to be established, and the diagnostic criteria for secondary glaucoma remain undefined. Furthermore, there is a scarcity of studies comparing diagnostic results and various treatment approaches, while existing research reveals significant individual variability across patient populations. The risk factors for these conditions are highly variable, and the underlying pathogenic mechanisms remain unclear. These gaps underscore the critical need for further research.

In conclusion, CMV infection of the anterior segment, particularly when complicated by secondary glaucoma, poses a formidable challenge to ophthalmology. Early recognition, accurate diagnosis and a comprehensive, individualized treatment plan combining antiviral therapy with aggressive IOP management are paramount. Continued efforts to refine diagnostic tools, improve antiviral and anti-glaucoma therapies, and clarify pathogenic mechanisms will be crucial for improving corneal endotheliitis and preventing the devastating consequences of secondary glaucoma.

## 8. Methods

The narrative review was conducted using PubMed, Web of Science, and Google Scholar databases, and included studies such as clinical cohort studies, case–control studies, observational studies, randomized controlled trials, and other relevant study types. Additionally, through relevant title and keyword searches, approximately 1176 articles were selected, including clinical studies, basic studies and reviews. Only English language reports during the years 1969–2026 were reviewed. Our article selection criteria were primarily based on the guidelines established by the Japan Corneal Endotheliitis Study Group in 2015 [34]. The inclusion criteria excluded studies involving diseases caused by HSV, VZV, CMV-induced uveitis, PSS, iridokeratitis, and primary glaucoma. The search strategy incorporated combinations of keywords related to CMV (e.g., “Cytomegalovirus,” “HHV-5,” “HCMV,” “Human betaherpesvirus 5,”) and terms related to corneal endotheliitis (e.g., “Corneal Endothelial Cell,” “Corneal Endothelium,” “inflammation,” “anterior segment”) and secondary glaucoma (e.g., “glaucoma,” “secondary”).

The novelty of this review lies in its thorough exploration of CMV endotheliitis and its associated secondary diseases, providing a detailed overview of the progression of both closely related diseases, including their clinical features, diagnostic approaches, management strategies, risk factors, and potential pathogenic mechanisms, with the hope of offering valuable clinical insights for future research. 

## Figures and Tables

**Table 1:** Summary of studies on the clinical features of CMV corneal endotheliitis.

Study	Sex (Male, n)	Age ^+^	No. of Eye Cases (Patients)	Corneal Edema	KPs	Mild Anterior Chamber Inflammation	IOP (mmHg) *
Koizumi et al. (2025)	11	72.2 ± 9.2	12 (12)	58.30%	Coin-shaped (75.0%); linear (41.7%); other (83.3%)	66.7%	19.7 ± 6.8 (5.0–31.0)
Mori et al. (2022)	31	69.1 ± 13.1 (27–87)	34 (34)	NR	Coin-shaped/linear (47.1%)	NR	22.4 ± 10.6; IOP > 21 (55.9%)
Kuo et al. (2022)	9	Median 62 (58–68)	15 (13)	60%	Coin-shaped (60%); pigmented (26.6%); mutton-fat (13.3%)	60%	IQR 42 (28–48); elevated IOP (93.3%)
Kobayashi et al. (2022)	7	Range 43–84	8 (8)	75%	Coin-shaped (87.5%); linear (12.5%)	87.5%	Range 27–60
Yokogawa et al. (2021)	2	Range 73–78	3 (3)	NR	Coin-shaped (33.3%); pigmented (66.7%)	NR	NR
Cheng et al. (2021)	48	54.1 ± 11.5	61 (61)	52.50%	Coin-shaped (37.7%)	63.9%	24.0 ± 9.2; IOP > 22 (49.1%)
Koizumi et al. (2015)	85	66.9 ± 10.9 (40–85)	109 (106)	73.40%	Coin-shaped (70.6%); linear (8.3%)	67.9%	19.6 ± 9.8; IOP > 22 (38.5%)
Kobayashi et al. (2012)	6	73.3	6 (6)	100%	Coin-shaped (50%); pigmented (100%)	50%	Range 15–43
Chee et al. (2012)	15	Median 57 (26–82)	21 (19)	75%	Linear fine/larger	95%	NR
Koizumi et al. (2008)	6	68.5 (51–83)	8 (8)	100%	Coin-shaped (100%); linear (50%)	50%	17.75 (9–32)
Chee et al. (2007)	8	49 (25–61)	12 (10)	90%	Fine/filiform/medium, pigmented/linear/circular	NR	40.85 ± 10.12

M, male; NR, not reported. ^+^ Mean Age ± SD (range), unless otherwise specified. * Mean IOP ± SD (range), unless otherwise specified.

## Data Availability

No new data were created or analyzed in this study.

## Supplementary Materials

The following supporting information can be downloaded at: https://www.mdpi.com/article/10.3390/pathogens15040371/s1, Table S1: Studies reporting the treatment of CMV corneal endotheliitis.
