# Supplementary material for: Cytomegalovirus Infection of the Anterior Segment: Corneal Endotheliitis and Secondary Glaucoma
Source: Pathogens. 2026 Mar 31;15(4):371. doi: 10.3390/pathogens15040371 (PMC13119229; doi:10.3390/pathogens15040371)
Supplement: Supplementary file 1 [file pathogens-15-00371-s001.zip › pathogens-4203059-supplementary/Supplementary table 1.pdf]

Supplementary table 1. Studies reporting the treatment of CMV corneal endotheliitis

Study	Country	No. of cases eyes (patients)	Prior history			Treatment regimen			
			Prior history of ocular disease or ocular hypertension	Prior history of immunosuppressive therapy	Prior history of ocular surgery	Antiviral		Steroids	Other treatment
						Systemic	Topical	Topical	
Koizumi et al. (2025)	Japan	12 (12)	IOP↑ (100%)	Steroids (75%)	NR		GCV gel	FML eye drops	
Deie et al. (2025)	Japan	1 (1)	IOP↑	NR	NR	VGCV	GCV	Steroids	
Bhamra et al. (2023)	Canada	9 (7)	AU, PSS	NR	Trab, Tube	VGCV		Pred	
Mori et al. (2022)	Japan	34 (34)	Glaucoma (94.1%)	Steroids (100%)	Cataract surgery (32.4%), corneal transplantation (8.8%), anti-glaucoma surgery (8.8%)	IV GCV, PO VGCV	GCV	Steroids eye drops	Anti-glaucoma
Kuo et al. (2022)	China	15 (13)	Glaucoma (86.7%)	Steroids	NR		GCV	Beta eye drops , FML	
Chen et al. (2022)	China	4 (4)	Endotheliitis	NR	NR		GCV	Pred, Beta,	Anti-glaucoma

								FML	
Kao et al. (2020)	China	1 (1)	NR	Pred, AZA	NR	PO VGCV	GCV		Anti-glaucoma
Cheng et al. (2021)	China	61 (61)	IOP↑ (100%)	Steroids (98.4%)	Glaucoma surgery (9.8%), corneal transplantation (11.5%), cataract surgery (32.8%), pseudophakia (32.8%)	IVT GCV, PO GCV			Antibiotic
Koizumi et al. (2015)	Japan	109 (106)	Glaucoma (65.1%)	Steroids (96.3%)	Glaucoma surgery (30.3%), corneal transplantation (25.7%), IOL implantation (55.0%)	GCV, VGCV	GCV, VGCV	Steroids eye drops	Anti-glaucoma
Ang et al. (2013)	Singapore	5 (5)	IOP↑	NR	Trab (40%), Tube (20%), pseudophakic (80%)	PO VGCV	GCV gel	Steroids, Pred	Antibiotic
Kobayashi et al. (2012)	Japan	6 (6)	IOP↑, secondary glaucoma, AU, PSS (66.7%), iridocyclitis (75%)	Steroids	Cataract surgery (75%)	IV GCV , PO GCV	GCV eye drops	Beta eye drops	
Chee et al. (2012)	Singapore	21 (19)	IOP↑	NR	Corneal graft (28.6%), pseudophakic (9.5%), glaucoma surgery	IV GCV, PO GCV, PO	GCV gel	Pred	NSAID

						VGCV			
Koizumi et al. (2008)	Japan	8 (8)	Uveitis, corneal endotheliitis	NR	PK (50%)	IV GCV	GCV, ACV	FML, Dex	Antibiotic
Shiraishi et al. (2007)	Japan	1 (1)	Corneal edema	Steroids	NR	IV GCV	GCV		
Chee et al. (2007)	Singapore	12 (10)	AU, PSS, glaucoma	Steroids (100%)	Trab (33.3%)	IV GCV, PO GCV, PO VGCV		Pred	

IOP↑, ocular hypertension or IOP elevation; NR, not reported; GCV, ganciclovir; FML, fluorometholone; VGCV, valganciclovir; AU, anterior uveitis; PSS, Posner–Schlossman syndrome; Trab, trabeculectomy; Tube, glaucoma drainage tube surgery; Pred, prednisolone; IV, intravenous; PO, oral; Beta, betamethasone; AZA, azathioprine; IVT, intravitreal; IOL, intraocular lens; ACV, acyclovir; NSAID, non-steroidal anti-inflammatory drug; PK, penetrating keratoplasty; Dex, dexamethasone.
